# Oral Gavage Delivery of Stable Isotope Tracer for In Vivo Metabolomics

**DOI:** 10.3390/metabo10120501

**Published:** 2020-12-08

**Authors:** Holden C. Williams, Margaret A. Piron, Grant K. Nation, Adeline E. Walsh, Lyndsay E. A. Young, Ramon C. Sun, Lance A. Johnson

**Affiliations:** 1 Department of Physiology, University of Kentucky College of Medicine, Lexington, KY 40536, USA; holden.williams@uky.edu (H.C.W.); maggie.piron@uky.edu (M.A.P.); grant.nation@uky.edu (G.K.N.); adeline.walsh@uky.edu (A.E.W.); 2 Sanders-Brown Center on Aging, University of Kentucky College of Medicine, Lexington, KY 40536, USA; 3 Department of Molecular and Cellular Biochemistry, University of Kentucky College of Medicine, Lexington, KY 40536, USA; lyndsay.young@uky.edu; 4 Department of Neuroscience, University of Kentucky College of Medicine, Lexington, KY 40536, USA; 5 Markey Cancer Center, University of Kentucky College of Medicine, Lexington, KY 40536, USA

**Keywords:** metabolomics, stable isotope, ^13^C-glucose, gavage, SIRM, Alzheimer’s disease, diabetes, brain metabolism

## Abstract

Stable isotope-resolved metabolomics (SIRM) is a powerful tool for understanding disease. Advances in SIRM techniques have improved isotopic delivery and expanded the workflow from exclusively in vitro applications to in vivo methodologies to study systemic metabolism. Here, we report a simple, minimally-invasive and cost-effective method of tracer delivery to study SIRM in vivo in laboratory mice. Following a brief fasting period, we orally administered a solution of [U-^13^C] glucose through a blunt gavage needle without anesthesia, at a physiological dose commonly used for glucose tolerance tests (2 g/kg bodyweight). We defined isotopic enrichment in plasma and tissue at 15, 30, 120, and 240 min post-gavage. ^13^C-labeled glucose peaked in plasma around 15 min post-gavage, followed by period of metabolic decay and clearance until 4 h. We demonstrate robust enrichment of a variety of central carbon metabolites in the plasma, brain and liver of C57/BL6 mice, including amino acids, neurotransmitters, and glycolytic and tricarboxylic acid (TCA) cycle intermediates. We then applied this method to study in vivo metabolism in two distinct mouse models of diseases known to involve dysregulation of glucose metabolism: Alzheimer’s disease and type II diabetes. By delivering [U-^13^C] glucose via oral gavage to the 5XFAD Alzheimer’s disease model and the Lep^ob/ob^ type II diabetes model, we were able to resolve significant differences in multiple central carbon pathways in both model systems, thus providing evidence of the utility of this method to study diseases with metabolic components. Together, these data clearly demonstrate the efficacy and efficiency of an oral gavage delivery method, and present a clear time course for ^13^C enrichment in plasma, liver and brain of mice following oral gavage of [U-^13^C] glucose—data we hope will aid other researchers in their own ^13^C-glucose metabolomics study design.

## 1. Introduction

Traditional techniques in targeted metabolomics provide quantitative analysis of metabolite pools within a selected network, thus offering a snapshot of overall changes in the metabolome. A more targeted understanding of a specific pathway can be obtained with a stable isotope labeled substrate, or isotopic tracer, which offers improved specificity for the pathways and metabolic enzymes involved; for example, identifying metabolites downstream of glucose metabolism/catabolism when ^13^C-glucose is use as the primary tracer.

Stable isotope-resolved metabolomics (SIRM) couples stable isotope tracing with nuclear magnetic resonance (NMR) and/or mass spectrometry (MS)-based analyses so that labeling patterns (isotopomers and isotopologues) of numerous metabolites can be determined for robust and large-scale reconstruction of metabolic networks [1]. This approach has been successfully applied to cultured cells, mouse models, tissue models and human patients [2,3,4]. SIRM has been applied most heavily in cancer research, where it has been used to elucidate alterations in metabolic networks associated with different cancers, as well as rare somatic mutations in isocitrate dehydrogenase [5,6,7], and germline lesions in fumarate hydratase [8,9,10] and succinate dehydrogenase [9,11,12].

With added insights through isotopic enrichment, SIRM builds on the foundational metabolomic methodologies that employ metabolite fractionation by either gas or liquid chromatography (GC; HPLC), or capillary electrophoresis (CE), followed by mass spectrometry detection [13]. An important consideration when designing a SIRM study is selecting the appropriate tracer substrate. Selection of the appropriate stable isotope (e.g., ^13^C, ^15^N, ^2^H, ^18^O, ^34^S) and substrate (e.g., glucose, lactate, glutamine, etc.) should rely upon the desired pathway to be investigated; and more selective analysis of specific nodes of interest within pathways can be resolved with specific number and position of stable isotopes. For example, [U-^13^C] glucose provides broad incorporation throughout central carbon metabolism pathways, while [1,2-^13^C] glucose provides specific resolution of oxidative and non-oxidative branches of the pentose phosphate pathway (PPP). A description of current stable isotope tracers and their respective utilization(s) is reviewed in detail by Jang, et al. [14]. The unique information gained through tracer metabolomics can be a powerful tool for biomarker identification and drug discovery, and aids in both disease profiling as well as mechanistic studies by providing a critical measure of the functional outcome from a given biological system.

While the majority of SIRM experiments are conducted in vitro, a variety of delivery methods have also been successfully applied to stable-isotope tracing in vivo. These include single or repeated injections of isotopic tracers via intraperitoneal (IP) injection, or direct injection into the circulation via intravenous (IV) infusion [15,16,17,18]. While these methods are cost-effective, they sacrifice the physiological absorption and metabolism for substrates that are normally ingested. Continuous infusions of the isotopically labeled substrate through cannulation is another common approach [19], as it offers the benefit of increased depth of labeling in more complex metabolic pathways and simpler interpretation as a result of achieving constant isotopic enrichment (i.e., isotopic steady state). However, the primary drawbacks of infusion methods are the significant invasiveness of the procedure, the high level of technical complexity required, and throughput limitations due to the long time periods required to test each individual animal. Alternatively, oral administration through ad libitum feeding of a diet in which the isotope has been incorporated has the advantages of being non-invasive, less stressful, and conducive to allowing the organism to reach isotopic steady state with ad libitum feeding over 16+ hours [20]. However, it is impossible to control for differences in the timing and amount of food intake in this ad libitum setting. The method also requires customized animal feed and relatively large amounts of isotopic label, and is thus quite expensive compared to other methods.

While no administration method is without drawbacks, isotope delivery via an oral gavage is an attractive strategy to mitigate several of the common limitations described above. The method is simple, minimally invasive, and requires low amounts of isotopic tracer. A handful of studies have employed an oral gavage to deliver ^13^C tracers, mainly fatty acids [21,22,23,24] and carbohydrates [25,26,27,28]. For example, one study examined ^13^C enrichment into glycogen following oral gavage of glucose [26], while another examined ^13^C enrichment in liver, small intestine, and skeletal muscle following oral gavages of a 1:1 mixture containing labeled fructose and unlabeled glucose or vice versa [28]. Here, we detail a simplified, cost-effective method of ^13^C isotope delivery based on the glucose tolerance test performed routinely in mouse models. This method produces robust isotopic distribution, with the added advantage of speed and consistency to improve rigor and reproducibility. We provide a detailed time course and labeling patterns for one of the most commonly used tracer substrates, [U-^13^C] glucose, in the plasma, liver and brain. Finally, we apply our method in two distinct mouse models of disease that feature metabolic components, demonstrating lower ^13^C-glucose enrichment in glycolysis in the Lep^ob/ob^ model of type II diabetes, and decreases in de novo neurotransmitter synthesis in the 5XFAD mouse model of Alzheimer’s disease.

## 2. Results

### 2.1. Experimental Workflow and Stable Isotope Tissue Distribution

**Figure 1:**
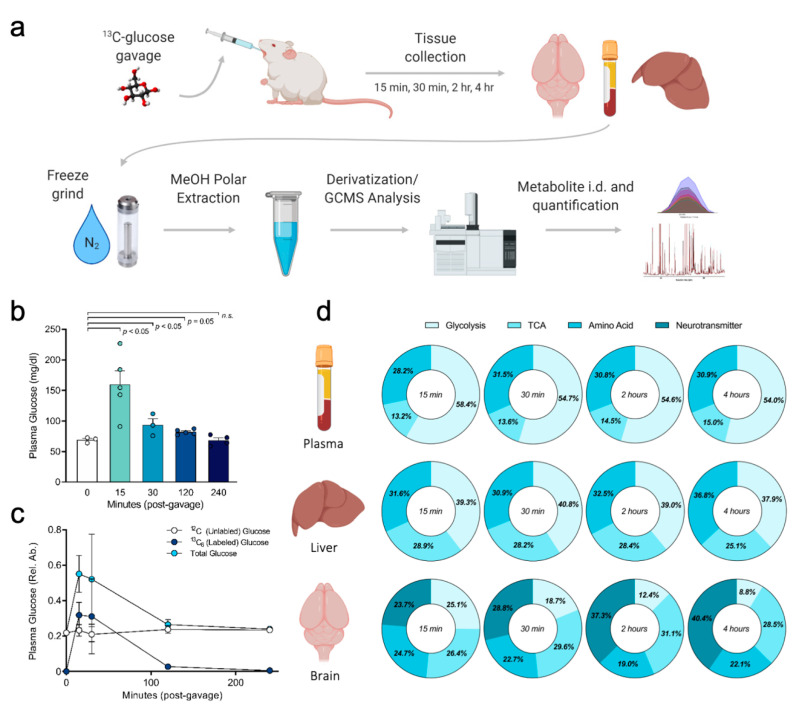
Experimental design, plasma glucose labeling and ^13^C pathway distribution analysis after oral gavage of [U-^13^C] glucose. (**a**) Schematic of metabolomics workflow for usage of stable isotope tracer in vivo delivered via oral gavage. Mice were orally gavaged [U-^13^C] glucose tracer (2 mg/kg bodyweight), euthanized 15 min, 30 min, 2 h, or 4 h post-gavage, tissues and plasma promptly collected and frozen in liquid nitrogen. Tissues were machine-ground under liquid nitrogen after which tissue (or plasma) polar metabolites extracted with MeOH, vacuum dried, derivatized, and analyzed via GCMS. Data interpreted using AMDIS and DExSI software for determining pathway enrichment. (**b**) Total plasma glucose was measured via colorimetric assay to determine plasma glucose concentration pre- and post-gavage (each time point represents one group of mice). (**c**) Plasma glucose measured via GCMS reveals labeled versus unlabeled glucose at each time point. (**d**) Pathway distribution analysis of ^13^C enrichment within specific metabolic pathways in plasma, liver, and brain. The average ^13^C fractional enrichment across representative metabolites from glycolysis, tricarboxylic acid (TCA) cycle, amino acids, and neurotransmitters were calculated, and shown as the percent of total ^13^C enrichment across these pathways for each tissue. Values shown are mean ± SEM (*n* = 3–5). Data analyzed by unpaired *t*-test, *p* values shown on graph (**b**).

For the current experimental workflow, universally labeled ([U-^13^C]) glucose was delivered to fasted mice (C57BL/6) via oral gavage (2 g/kg bodyweight). At select time points (15 min, 30 min, 2 h, or 4 h post-gavage), tissues were harvested and prepared for analysis by gas chromatography mass spectrometry (GCMS) (Figure 1a). Total plasma glucose concentrations followed an expected time course following the ^13^C-glucose gavage, with a peak concentration around 15 min and a return to baseline (fasting) concentrations after 4 h (Figure 1b). Quantification of ^13^C-glucose in the plasma showed that by 15 and 30 min, >50% of plasma glucose was fully labeled (^13^C_6_; m+6), and by 4 h post-gavage <5% of circulating glucose was fully labeled (Figure 1c). We next examined ^13^C enrichment of representative metabolites within pathways of glycolysis, TCA, amino acid and neurotransmitter synthesis in the plasma, liver and brain of mice gavaged with [U-^13^C] glucose. The pathway distribution analysis (Figure 1d) shows the average enrichment per pathway, represented as donut charts. Distribution of ^13^C among metabolic pathways varied greatly by tissue, with the highest incorporation into glycolysis in plasma and liver, which was prevalent at all time points (Figure 1d, first and second row). However, ^13^C incorporation in brain 15 min post-gavage was distributed relatively equally into glycolysis, TCA cycle, amino acid, and neurotransmitter synthesis pathways, while neurotransmitter synthesis was most prominent at later time points (Figure 1d, third row). The pathway distribution of ^13^C within tissues also shifted over time, for example in brain, where the percent of ^13^C found in neurotransmitters (glutamate, aspartate, γ-aminobutyric acid (GABA), and pyroglutamic acid) increased from 23.7% at 15 min to 40.4% at 4 h (Figure 1d, third row). The time course of fractional enrichment in plasma (Figure S1, Supplementary Material) highlights ^13^C labeling in metabolites at 15 min, 30 min, 2 h, and 4 h post-gavage. Fractional enrichment in the labeled isotopologues of glucose show that fully labeled, glucose (m+6) is the most abundant at 15 and 30 min, accounting for nearly 50% of total glucose, and decreases to less than 10% after 2 h (Figure S1a). Roughly 20% of pyruvate and lactate in the plasma was fully labeled at 15 and 30 min and decreases thereafter, while citrate and other metabolites associated with the TCA cycle show a slower, less prevalent enrichment that peaks at 2 h (Figure S1b–d). Together, these data show that the described method for ^13^C substrate administration via oral gavage reliably achieves sufficient incorporation into multiple central carbon pathways (glycolysis, TCA cycle, and amino acid metabolism pathways) in multiple tissues.

### 2.2. Brain Metabolites Display Varying Patterns of ^13^C Labeling

**Figure 2:**
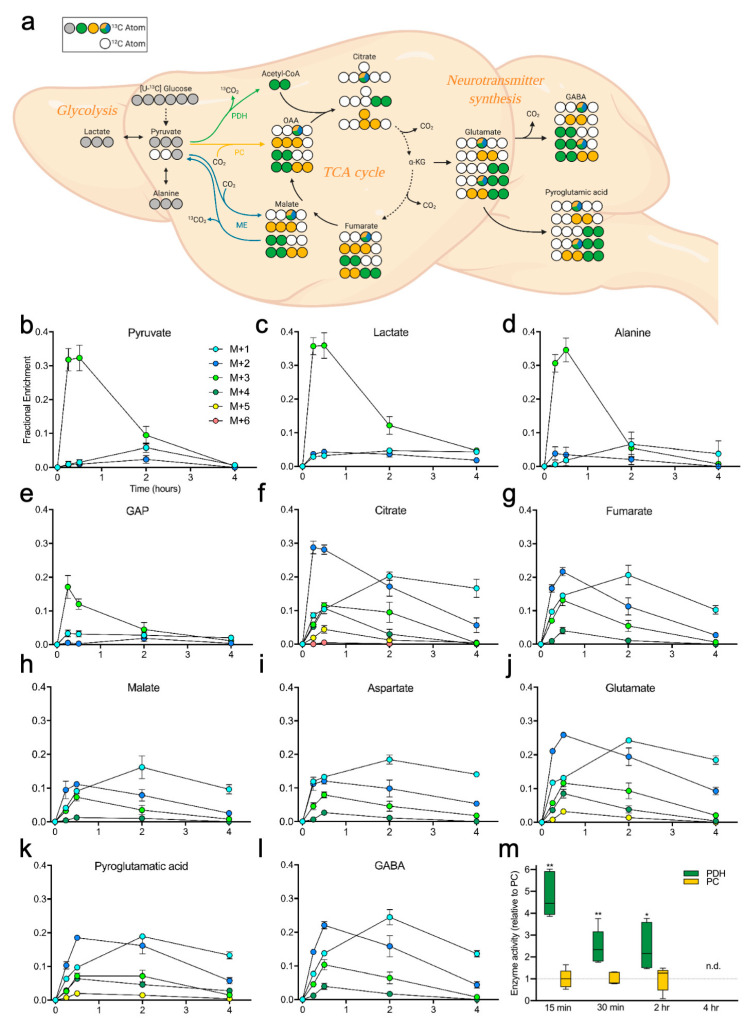
Carbon tracing in the brain after oral gavage of [U-^13^C] glucose. (**a**) Example diagram tracing of glucose carbon through glycolysis, TCA cycle, and neurotransmitters in whole-brain tissue from mice receiving oral gavage of [U-^13^C] glucose (not all possible labeled isotopologues are shown). Carbons are color coded based on the pathway and/or enzyme from which the carbon was derived. Grey circles: ^13^C from glycolysis; green: ^13^C from pyruvate dehydrogenase (PDH); yellow: ^13^C from pyruvate carboxylase (PC); blue, yellow, green tricolor: ^13^C from malic enzyme (ME), reentry via ME, PC, or PDH; oxaloacetate (OAA); α-ketoglutarate (α-KG); γ-aminobutyric acid (GABA). (**b**–**l**) Fractional enrichment of ^13^C from glucose over time in the labeled isotopologues of pyruvate, lactate, Ala, glyceraldehyde phosphate (GAP), citrate, fumarate, malate, Asp, Glu, pyroglutamic acid, and GABA, respectively. (**m**) Surrogate enzyme activities were calculated for PDH (citrate m+2/pyruvate m+3) and PC (citrate m+3/pyruvate m+3) in the brain, showing their respective contributions toward metabolism of [U-^13^C] glucose (via pyruvate) into the TCA cycle over time. Values shown are mean ± SEM (*n* = 4–5) (**b**–**m**). Data analyzed by unpaired *t*-tests (**m**). * *p* < 0.05; ** *p* < 0.01.

We next examined specific labeling patterns of ^13^C incorporation in the brain, with a specific focus on glycolytic and TCA cycle intermediates as well as neurotransmitter synthesis (Figure 2a). The labeling of TCA cycle intermediates can provide information regarding contributions from the enzymes pyruvate dehydrogenase (PDH), pyruvate carboxylase (PC), and malic enzyme (ME), which govern pyruvate entry to the TCA cycle (Figure 2a, color coded). PDH liberates one carbon atom from pyruvate as CO_2_ and incorporates two carbons (via acetyl-CoA), while PC incorporates all three carbons from pyruvate. The reversible ‘malic’ enzyme reaction (malate dehydrogenase-decarboxylating) can generate singly labeled (m+1) isotopologues via conversion of m+1 pyruvate and CO_2_ to form m+1 malate (and subsequently m+1 oxaloacetate via malate dehydrogenase), that can be further incorporated into other TCA cycle intermediates [20]. Fractional enrichment of specific metabolites isolated from whole brain tissue over time are shown in Figure 2b–l. Metabolites associated with glycolysis (glyceraldehyde phosphate [GAP], pyruvate, lactate, and alanine) displayed the highest enrichment at 15 and 30 min (Figure 2b–d). Conversely, the labeling in TCA cycle intermediates generally plateaued at 2 h and/or began to decline by 4 h (Figure 2f–i). Although technical limitations hinder measuring oxaloacetate (OAA) directly, aspartate fractional enrichment (Figure 2i) reflects that of OAA due to its equilibrium with aspartate [29]. Additionally, two abundant neurotransmitters (pyroglutamic acid and GABA) displayed enrichment patterns similar to their precursor for synthesis, glutamate, with peak enrichment at 2–4 h post-gavage (Figure 2j–l). Finally, labeling patterns among pyruvate and citrate can be used as surrogates to estimate enzyme activity of PDH and PC [21], calculated as (citrate m+2/pyruvate m+3) and (citrate m+3/pyruvate m+3), respectively. Our data show increased activity of PDH relative to PC 15 min post-gavage, with decreasing contributions of both enzymes over time reflecting the passage of the ^13^C-glucose bolus through the TCA cycle (Figure 2m). Brain time course data available in Supplementary File S1.

### 2.3. Measuring Glucose Metabolism in an Alzheimer’s Disease Model

**Figure 3:**
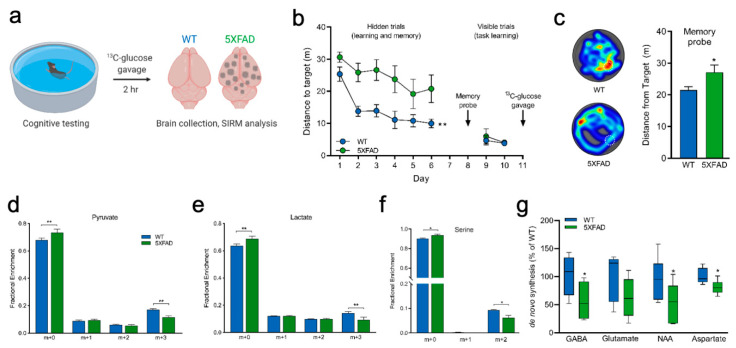
Cerebral glucose metabolism in the 5XFAD Alzheimer’s disease mouse model. (**a**) Experimental paradigm depicting the cognitive testing of 5XFAD and WT mice followed by an oral gavage of [U-^13^C] glucose, 2 h after which brain tissue was collected for stable isotope-resolved metabolomics (SIRM). (**b**) Behavioral testing confirms cognitive impairment of the 5XFAD relative to WT mice, as shown by decreased latency (time to reach hidden platform) in the Morris water maze. (**c**) Left: Representative heat maps showing swim paths of 5XFAD and WT during the memory probe of the water maze (dotted white circle indicates location of the hidden platform; red color indicates areas of greater activity). Right: 5XFAD mice show a significantly increased distance from the target during the memory probe, indicating an impairment in long-term memory. (**d**–**g**) Fractional labeling patterns of pyruvate (**d**), lactate (**e**), and Ser (**f**) show ^13^C enrichment in brain tissue 2 h after oral gavage of [U-^13^C] glucose. (**g**) Percentage of ^13^C enrichment in γ-aminobutyric acid (GABA), Glu, n-acetylaspartate (NAA), and Asp indicate de novo synthesis of neurotransmitters from glucose (shown as percent of average labeled isotopologues in WT). Values shown are mean ± SEM (*n* = 6–7). Data analyzed by repeated measures ANOVA (**b**), unpaired *t*-test (**c**,**g**), and multiple *t*-tests (**d**–**f**). * *p* < 0.05; ** *p* < 0.01.

In order to illustrate a potential application of this oral gavage technique, we examined how cerebral glucose metabolism is affected in the 5XFAD mouse model of Alzheimer’s disease [30]. Immediately following their final cognitive testing session, 5XFAD mice and age- and sex-matched WT controls were given an oral gavage of [U-^13^C] glucose and brain tissue collected for metabolomic analysis after 2 h (Figure 3a). The commonly used 5XFAD model of Alzheimer’s disease shows early and significant cognitive deficits, as evidenced by decreased performance in a water maze test of spatial learning and memory (Figure 3b,c). Interestingly, brains from 5XFAD mice exhibited decreased fractional enrichment of fully labeled pyruvate and lactate (m+3), indicative of a reduction in glycolysis relative to WT mice (Figure 3d,e), and further demonstrated by the reduced labeling in serine denoted by the decreased m+2 isotopologue (Figure 3f). Additionally, glucose-derived synthesis of the neurotransmitters GABA, N-acetylaspartate (NAA), and aspartate were significantly reduced in the 5XFAD model relative to the WT controls (Figure 3g).

**Figure 4:**
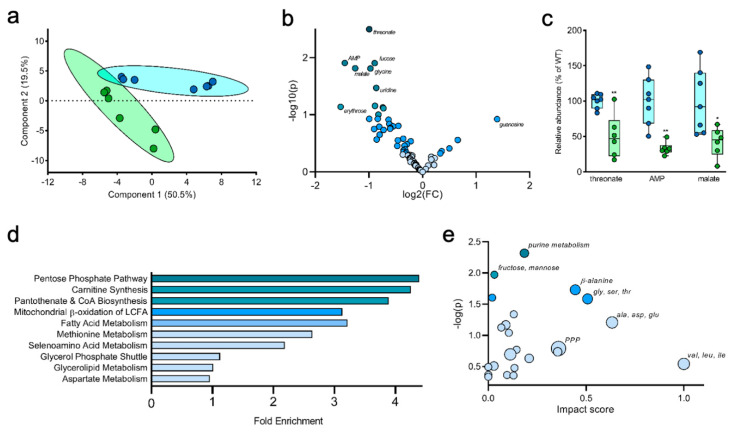
Non-tracer metabolomics analysis. (**a**) Principle component analysis distinguishes between the metabolic profile of 5XFAD brain from WT based on 80 metabolites identified by GCMS analysis. (**b**,**c**) A change volcano plot separates metabolites based on degree of change versus statistical significance among 5XFAD and WT (**b**), and select metabolites identified in Figure 4b are further highlighted in Figure 4c by direct comparison of their relative abundance. (**d**) Fold-enrichment analysis compares the major metabolic pathway differing between 5XFAD and WT. (**e**) Pathway impact analysis provides a multi-dimensional illustration of metabolic pathways based on pathway enrichment analysis, pathway topology analysis, and statistical significance. Values shown are mean ± SEM (*n* = 6–7) (**c**). Data analyzed by unpaired *t*-test (**c**). * *p* < 0.05; ** *p* < 0.01.

Importantly, in addition to the quantification and interpretation of stable isotope enrichment, the datasets generated with this method can also be mined for information from total metabolic pool sizes (regardless of labeling). To generate a more global view of metabolism in the 5XFAD brain, we quantified the relative pool size of ~80 readily identified metabolites and analyzed these data using Metaboanalyst [31]. A principal component analysis showed that the 5XFAD and WT mice have distinct metabolic profiles (Figure 4a), while a volcano plot highlighted several specific metabolites that were uniquely altered in the 5XFAD brain (Figure 4b). The most substantially altered metabolites included threonate, adenosine monophosphate (AMP), and malate, which were all significantly reduced in the 5XFAD brain compared to WT (Figure 4c). To determine which metabolic pathways were most affected, we performed a pathway enrichment analysis, which highlighted the pentose phosphate pathway (PPP), carnitine synthesis, pantothenate and CoA biosynthesis, mitochondrial beta oxidation, and fatty acid metabolism as the most significantly altered pathways between 5XFAD and WT mice (Figure 4d). Finally, a pathway impact analysis showed several major differences between WT and 5XFAD brain samples, highlighting changes in the concentration of metabolites considered key nodes within their pathways. Pathways highlighted by this impact analysis included hexose metabolism (fructose, mannose), purine metabolism, PPP, and several amino acid processing pathways (β-alanine; glycine, serine and threonine; alanine, aspartate and glutamate; valine, leucine and isoleucine) (Figure 4e).

### 2.4. Liver Metabolites Display Varying Patterns of ^13^C Labeling

**Figure 5:**
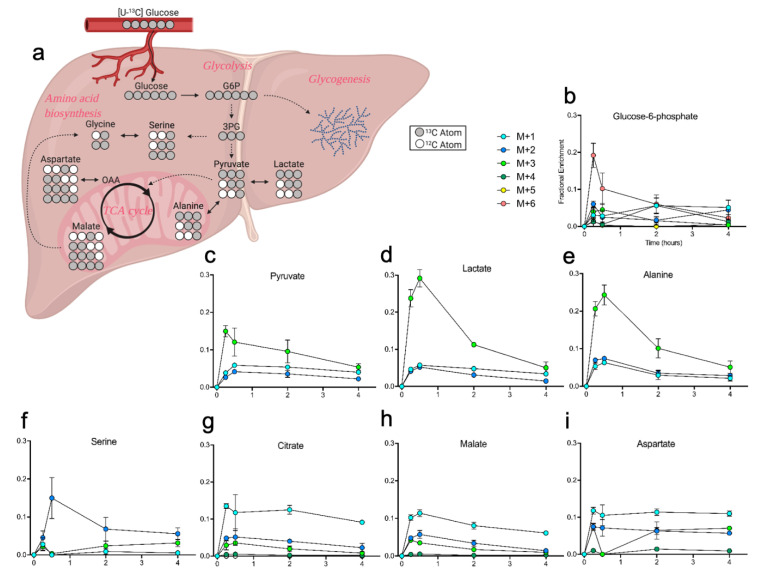
Carbon tracing in the liver after oral gavage of [U-^13^C] glucose. (**a**) Diagram tracing of glucose through central carbon metabolism pathways in the liver. Not all possible labeled isotopologues are shown. Grey circles: ^13^C; white circles: ^12^C. (**b**–**i**) Fractional enrichment of ^13^C from glucose over time in the labeled isotopologues of glucose-6-phosphate, pyruvate, lactate, Ala, Ser, citrate, malate, and Asp, respectively. Values shown are mean ± SEM (*n* = 4–5).

We also examined ^13^C incorporation in the liver, detecting enriched metabolites across all time points post-gavage. In the liver, glucose that enters glycolysis is actively further metabolized in pathways such as the TCA cycle and amino acid biosynthesis (Figure 5a). Fully labeled (m+6) glucose-6-phosphate (G6P) was the dominant isotopologue 15 min post-gavage, and the presence of measurable fractions of other labelled G6P isotopologues suggests a significant amount of ^13^C scrambling (Figure 5b). Enrichment of pyruvate showed a steady decline in labeling over time after the predominate isotopologue m+3 peaked at 15 min post-gavage (Figure 5c). Lactate and alanine labeling were more robust than pyruvate, likely a reflection of two processes where the interconversion of lactate- and alanine-pyruvate (Cori and Cahill cycles) divert pyruvate for extrahepatic metabolism (Figure 5d,e) [32]. Serine m+2 increased in abundance from 15 to 30 min post-gavage, likely contributed from glycine formed through ^13^C cataplerosis from the TCA cycle (Figure 5f). Citrate and malate showed direct labeling of TCA intermediates, which are used to generate amino acids like aspartate, and as such they displayed similar fractional labeling patterns (Figure 5g–i). Finally, using our previously established method of glycogen quantitation [33,34], we observed dynamic glycogen enrichment in the liver and brain. Liver glycogen enrichment peaked at 30min, followed by a slow but steady decline until 4 h post gavage, while a similar pattern was observed in brain (Figure S2, Supplementary Materials). Liver time course data available in Supplementary File S1.

### 2.5. Tracing Glucose Metabolism in a Mouse Model of Type II Diabetes

**Figure 6:**
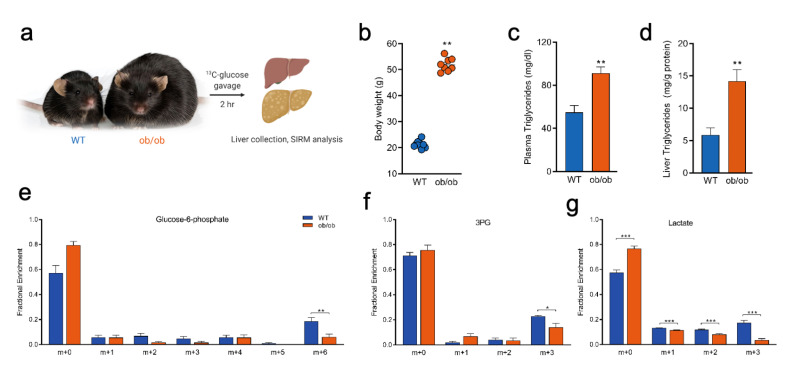
Liver glucose metabolism in the *Lep*^ob/ob^ (ob/ob) obesity and type II diabetes mouse model. (**a**) Experimental paradigm depicting ob/ob and WT mice receiving oral gavage of [U-^13^C] glucose, 2 h after which tissues and plasma were collected. (**b**) Bodyweight measurement shows greater body mass of ob/ob mice relative to WT. (**c**,**d**) Triglyceride levels measured via colorimetric assay reveals increased plasma (C) and liver (**d**) triglycerides levels in ob/ob mice. (**e**–**g**) Fractional labeling patterns in glucose-6-phosphate (**e**), 3-phosphoglycerate (3PG; **f**), and lactate (**g**). Values shown are mean ± SEM (*n* = 4–5). Data analyzed by unpaired *t*-test (**b**–**d**) and unpaired, multiple *t*-tests (**d**-**e**). * *p* < 0.05; ** *p* < 0.01; *** *p* < 0.001.

To illustrate the potential application of this method in a second disease model system, we used leptin deficient ob/ob mice (Lep^ob/ob^), a common mouse model of obesity and type II diabetes [35]. We confirmed several phenotypic characteristics of the model and then employed our method of stable isotope tracer delivery to highlight potential changes in hepatic glucose metabolism (Figure 6a). Lep^ob/ob^ mice reliably demonstrate several features of type II diabetes, including increased adiposity, hyperlipidemia, and fatty liver, as reflected by increased bodyweight, plasma triglycerides, and hepatic triglyceride content measurements (Figure 6b–d). To examine specific metabolic perturbations in Lep^ob/ob^ mice, we harvested liver tissue 2 h following gavage of [U-^13^C] glucose. The fractional enrichment distribution in several glycolytic intermediates were significantly altered between Lep^ob/ob^ and WT mice. Specifically, Lep^ob/ob^ showed decreased m+6 G6P, m+3 3-phosphoglyercate (3PG), and all labeled isotopologues of lactate (m+1, m+2, m+3; Figure 6d,e). Taken together, these results suggest decreased glucose enrichment in downstream metabolic products from Lep^ob/ob^ mice relative to WT, 2 h after oral gavage of [U-^13^C] glucose.

## 3. Discussion

SIRM analysis in vitro has become a widespread methodology to allow a more targeted interrogation of perturbed metabolic pathways [36]. More recent advances have allowed the delivery of stable isotope tracers to ex vivo tissue slices [37], small animals and even human patients. In vivo SIRM remains a challenging task despite being a very desirable workflow and is therefore a technique still limited to a select number of laboratories around the world. In order to facilitate wider adoption of this valuable in vivo methodology, physiologically relevant cost-effective methods for in vivo metabolomics analyses are needed. In the current study, we describe a simple, affordable and robust method of in vivo SIRM in which [U-^13^C] glucose is delivered to mice via an oral gavage. We provide a detailed time course of ^13^C incorporation into the plasma, liver and brain of mice, and further demonstrate the utility of this technique by highlighting metabolic changes in two commonly used mouse models of Alzheimer’s disease and type II diabetes.

In vivo SIRM is inherently complex, as the tissue metabolome is an interconnected myriad of de novo synthesized metabolites and circulating metabolites as products of systemic metabolism. Methods of tracer delivery via IV and IP injections or microdialysis may be more beneficial for use of tracers such as fatty acids or triglycerides, which require longer term metabolic processing for incorporation into downstream metabolic pathways within peripheral tissues [38]. However, because direct infusions into circulation or tissues bypass normal gastrointestinal processing and absorption, some physiological relevance is sacrificed. While single injections avoid the high cost associated with continuous infusions, many preliminary trials are often necessary to obtain the optimal time point after the one-time bolus. Furthermore, the stress due to the inherent invasiveness of such delivery methods can further complicate results.

Other less-invasive methods currently used for administering tracers in vivo include various modes of dietary incorporation ad libitum (normal dietary supplementation and liquid diet). These methods, also detailed by Garcia, et al. [38], retain the physiological relevance of enteral ingestion lost through direct infusions. Unfortunately, they share the common problems of low throughput, access to unique expertise, and most importantly high cost (often upwards of over $300 per mouse). For example, the liquid diet isotopic tracer delivery method allows SIRM analysis at isotopic steady state beyond central carbon metabolism, is minimally invasive, and permits ad libitum consumption of tracer [20]. However, there are two major biological drawbacks of this technique. First, the long (14–24 h) enrichment time allows for extensive isotopic exchange between de novo synthesized and circulating metabolites and results in isotopic scrambling in host tissues, thus substantially complicating interpretation. Second, the method is limited to animals with normal eating habits, i.e., not sick or behaviorally abnormal. For example, animals that carry xenografted tumors or overt neurological manifestations cannot be studied using the liquid diet.

An ideal in vivo delivery method would be cost effective, minimally invasive, with improved throughput; and we designed the gavage method described herein with all three parameters in mind. While dosing can easily be modified using the gavage method, the current experimental design was based on the principle of the commonly used oral glucose tolerance test (OGTT) (2 g of glucose per kg of bodyweight (40 mg/animal of 20 g)). This dose is based on clinical tests routinely performed on humans [39], and is commonly used in mouse studies of obesity, insulin resistance and diabetes [40]. The dietary glucose bolus is delivered via gavage without trauma or anesthesia [41], and can easily allow analysis of upwards of 50 animals per day (this study utilized 30 animals over the course of six hours). Thus, we anticipate that this delivery method will allow greater facilitation and utilization of in vivo SIRM in the research community. As this method is based off the OGTT routinely employed in the clinic, we anticipate that similar time course studies can be conducted in human subjects. Indeed, tracing of ^13^C substrates has been applied by several groups in human studies, as nicely reviewed by *Garcia,* et al. [38]. A primary drawback for this approach, however, is that while multiple plasma samples can easily be harvested and analyzed, analysis of human tissue can only occur ex vivo (as opposed to a ^13^C NMR based method) and thus requires collection of excised tissue (ex. surgically resected tumors or biopsies).

Here, we performed a time course of enrichment from [U-^13^C] glucose in multiple tissues, highlighting windows of isotopic distribution and utility to simultaneously interrogate multiple organs from a single mouse. We demonstrate the broad applicability of this method in commonly used mouse models of Alzheimer’s disease (5XFAD) and type II diabetes (Lep^ob/ob^). We showed how decreased glucose metabolism influences de novo neurotransmitter synthesis in the 5XFAD mouse model which may correlate with cognitive deficits. The 5XFAD mouse model has been shown to display glucose hypometabolism, as detected by a decreased FDG-PET signal [42]. Glucose hypometabolism is a common feature of AD that can occur in tandem with reduced cerebral blood flow thereby affecting metabolism by limiting oxygen availability to certain regions [43]. As a cell’s metabolic activity (and thus the pattern of ^13^C enrichment detected in an experimental setting) is clearly affected by availability of oxygen for mitochondrial respiration, researchers should consider potential changes in oxygen consumption in their disease model of choice. For example, reduced mitochondrial respiration has been reported in the brain of 5XFAD mice [44], and it is possible that these decreases in oxygen consumption are reflected in our data showing decreased ^13^C labeling of neurotransmitters synthesized from TCA cycle intermediates in 5XFAD mice compared to WT controls. Decreases in glycolysis shown in the Lep^ob/ob^ model are likely attributed to hampered insulin sensitivity and may also result in part from the increased hepatic and systemic adiposity. Interestingly, we did not observe striking differences in fractional labeling patterns of TCA cycle intermediates with either mouse model, which is surprising given the known mitochondrial dysfunction associated with both the 5XFAD and Lep^ob/ob^ mice [45,46]. However, metabolism of glucose by the TCA cycle may be further resolved with intermediary time points between 30 min and 2 h as presented herein. Furthermore, as our measurements were taken at early post-prandial time points, we did not observe any signs of liver gluconeogenesis in plasma or liver. This is likely a reflection of post-prandial activation of pathways of glucose storage (e.g., glycogenesis, as shown in Figure S2). However, analysis of later time points would likely offer more insight into the gluconeogenic role of the liver.

It should also be noted that any potential differences in TCA cycle intermediates inherent to either mouse model could be obscured given the rapid onset of tissue hypoxia, which occurs just seconds post-mortem. Although some degree of tissue hypoxia is likely unavoidable without a microwave fixation system, it can be mitigated with expeditious tissue collection. Thus, despite the relatively short period (less than 15 sec) between euthanasia and freezing of tissue employed in this study, artifactual changes in metabolism due to tissue hypoxia remain a concern with this and many other in vivo metabolomic approaches.

Altogether, we detected 80 metabolites across more than 10 metabolic pathways, of which 15 metabolites involved directly in central carbon metabolism displayed various isotopic distributions of ^13^C enrichment from [U-^13^C] glucose. When applied to animal models of disease, the extensive information made available through this method affords substantial potential in characterizing metabolic networks affected by diseases that may lead to elucidating metabolic targets for therapeutic interventions. While the GCMS employed in the current study is relatively affordable, it is limited in the number of metabolites detected. A more robust mass spectrometer could be used to assess isotopic enrichment in additional pathways such as the PPP and nucleotide metabolism.

Another limitation is that while we assume minimal invasiveness through previously established plasma corticosterone levels after gavage [41], we still anticipate an increase in stress through handling of the mice. However, based on this literature, we expect that the stress level is much lower compared to IV/IP injection and/or anesthetized animals—and can be further mitigated by conditioning the mice through repeated handling. While this delivery method has great potential to uncover much of the in vivo metabolome, tissue heterogeneity presents concerns of discerning specific contributions from distinct subpopulations of cells. Performing metabolomics on cell-sorted populations might alleviate such concerns, but current techniques are encumbered with metabolism-altering effects of sorting [47]. Imaging techniques like matrix assisted laser desorption/ionization (MALDI) and desorption electrospray ionization (DESI) might provide an alternative solution for tissue heterogeneity through spatial resolution of metabolites in prepared tissues. Application of MALDI or DESI in tissue obtained from an isotopic enriched animal could provide unique insight into metabolism at the micro-region and/or cell population level, representing an important and exciting next step to better understanding the in vivo metabolome.

## 4. Materials and Methods

### 4.1. Animals

All animal procedures were performed in accordance with institutional regulations and the institutional animal care and use ethics committee at the University of Kentucky College of Medicine (Protocol #2016-2569; approval date: 1 March 2020). Female wild type (WT) mice of C57BL/6 background (14 weeks old), ordered from the Jackson Laboratory (Bar Harbor, ME, USA) were housed in a climate-controlled environment with a 14/10-h light/dark cycle with water and regular chow diet ad libitum. Sex- and age-matched Lep^ob^ (ob/ob), ordered from the Jackson Laboratory (Bar Harbor, ME, USA), were used as a liver glucose metabolism comparison to WT mice. Sex- and age-matched homozygous 5XFAD, ordered from the Jackson Laboratory (Bar Harbor, ME, USA), mice were used for behavioral testing and metabolomics analysis as a comparison to WT mice.

### 4.2. Gavage of [U-^13^C] Glucose Solution

[U-^13^C] Glucose (Cambridge Isotope Laboratories, Tewksbury, MA, USA) was dissolved in ddH_2_O (Millipore Milli-Q, Bedford, MA, USA) based on the average mouse cohort bodyweight (2 g [U-^13^C] glucose/kg bodyweight), based on diabetic glucose tolerance testing. Mice were fasted for 2–4 h then administered a 250 µL volume of glucose solution via oral gavage. Blood and tissues (brain and liver) were collected 15 min, 30 min, 2 h, or 4 h post-gavage, detailed below. Blood and tissue were also collected from mice following starvation period (no gavage) and 4 h following either saline or non-labeled glucose gavage for use as controls for ^13^C labeling and gavage protocol.

### 4.3. Plasma and Tissue Collection

Following respective post-gavage incubation periods, mice underwent cervical dislocation and decapitation after which brain and liver were promptly removed and frozen in liquid nitrogen. Blood was collected via trunk bleed into tube containing 0.5M EDTA (Thermo Fisher Scientific, Waltham, MA, USA) and kept on ice until plasma was separated via centrifugation at 1300 × g for 10 min at 4 °C. The plasma layer (supernatant) was collected in two aliquots then flash frozen in liquid N_2_ until derivatization protocol, detailed below. Following prompt removal of brain and liver, tissues were washed in PBS (Bio-Rad, Hercules, CA, USA) twice, then ddH_2_O and quickly blotted dry before submersion in liquid N_2_, and stored at −80 °C until further processing.

### 4.4. Glucose Colorimetric Assay

Blood glucose concentrations were measured in plasma samples from WT mice using a glucose colorimetric assay (Cayman Chemical, Ann Arbor, MI, USA). The procedure was conducted according to manufacturer’s protocol. Briefly, plasma samples were thawed on ice then a volume of 15 µL per sample and manufacturer’s standards were added to a 96-well plate followed by addition of 85 µL assay buffer. Following addition of enzyme mixture, 100 µL, the plate was incubated for 10 min at 37 °C. After which a plate reader was used to read the absorbance at 510 nm and sample absorbances were compared with the standard curve to determine concentrations.

### 4.5. Triglyceride Assay

Blood and liver triglyceride concentrations were measured in WT and Lep^ob/ob^ using a triglyceride colorimetric assay (Cayman Chemical, Ann Arbor, MI, USA). Plasma samples were thawed on ice and diluted 1:9 with sodium phosphate assay buffer. Liver samples were thawed on ice and approximately 150 mg was finely minced and homogenized in 1 mL of diluted NP40 reagent containing proteinase inhibitors provided by the manufacturer. The remaining procedure was conducted according to manufacturer’s protocol. Briefly, liver samples were centrifuged at 10,000× *g* for 10 min at 4 °C, then the supernatant was diluted 1:4 with NP40. A volume of 10 ul was used for samples and triglyceride standards followed by addition of reaction mixture and 15 min incubation at room temperature. The absorbance at 540 nm was read using a plate reader at 540 nm and sample absorbances were compared with the standard curve to determine concentrations.

### 4.6. Sample Preparation for GCMS Analysis

All tissues were handled on liquid nitrogen when removed from cryostorage at −80 °C. Brain and liver samples were removed from cryostorage and pulverized to 10 µm particles in liquid N_2_ using a Freezer/Mill Cryogenic Grinder (model 6875D, SPEX SamplePrep, Metuchen, NJ, USA). Eighty microliters of plasma were extracted with 80 µL HPLC-grade MeOH (containing 40 µM L-norvaline [Sigma-Aldrich, St. Louis, MO, USA] for internal standard; Sigma-Aldrich, St. Louis, MO, USA). Approximately 60 mg of powdered tissue was removed with a micro spatula to a new tube and extracted with 50% MeOH (20 µM L-norvaline). Following addition of MeOH, mixtures were placed on ice for 20 min and briefly vortexed at 5 min intervals. Following centrifugation at 24,000× *g* for 10 min at 4 °C the aqueous phase containing polar metabolites was isolated to a separate tube, the resulting pellet briefly dried at 10^−3^ mBar using a SpeedVac (Thermo Fisher Scientific, Waltham, MA, USA) to evaporate remaining MeOH then reconstituted in RIPA buffer (Sigma-Aldrich, St. Louis, MO, USA) followed by BCA Protein Assay Kit (Pierce; Thermo Fisher Scientific, Waltham, MA, USA) for protein concentration. The polar metabolites were dried at 10^−3^ mbar followed by derivatization. The dried polar metabolite pellet was derivatized by a two-step methoxyamine protocol first by addition of 50 µL methoxyamine HCl (Sigma-Aldrich, St. Louis, MO, USA) in pyridine (20 mg/mL; Sigma-Aldrich, St. Louis, MO, USA) followed by 60 min dry heat incubation at 60 °C. Samples were then transferred to v-shaped glass chromatography vials (Agilent Technologies, Santa Clara, CA, USA) and sequential addition 80 µL N-methyl-trimethylsilyl-trifluoroacetamide (MSTFA; Thermo Fisher Scientific, Waltham, MA, USA) followed by 60 min dry heat incubation at 60 °C. The samples were allowed to cool to room temperature then analyzed via GCMS.

### 4.7. Glycogen Preparation for GCMS Analysis

Following removal of polar metabolites by 50% MeOH, the biomass fraction containing protein and glycogen were first resuspended in ddH_2_O followed by the addition of equal part 2N hydrochloric acid (HCl; Sigma-Aldrich, St. Louis, MO, USA). Samples were vortexed thoroughly and incubated at 95 °C for 2 h to allow the hydrolysis of glycogen to single glucose units. The reaction was quenched with 100% MeOH with 40 μM L-norvaline, samples were incubated on ice for 30 min, and the supernatant collected after centrifugation at 21,130× *g* at 4 °C for 10 min. The collected supernatant was subsequently dried by vacuum centrifuge at 10^−3^ mBar followed by derivatization similar to above.

### 4.8. GCMS Quantitation

GCMS (Agilent Technologies, Santa Clara, CA, USA) protocols were similar to those described previously [48,49] except a modified temperature gradient was used for GC: Initial temperature was 130 °C, held for 4 min, rising at 6 °C/min to 243 °C, rising at 60 °C/min to 280 °C, held for 2 min. The electron ionization (EI) energy was set to 70 eV. Scan (m/z: 50–800) and full scan mode were used for metabolomics analysis. Mass spectra were translated to relative metabolite abundance using the Automated Mass Spectral Deconvolution and Identification System (AMDIS) software matched to the FiehnLib metabolomics library (available through Agilent) for retention time and fragmentation pattern matching with a confidence score of >80 [13,50,51,52]. Quantitation was performed and corrected for natural abundance using the Data Extraction for Stable Isotope-labelled metabolites (DExSI; https://github.com/DExSI/DExSI) with a primary ion and at least 2 or more matching qualifying ions. Relative abundance was corrected for recovery using L-norvaline and adjusted to protein input from BCA measure to correct for any differences in tissue quantity used for extraction.

### 4.9. Metabolomics Data Analysis

Fractional enrichment of each metabolite was calculated as the relative abundance of each isotopologue relative to the sum of all other isotopologues. Pathway distribution analysis was determined for glycolysis, TCA cycle, amino acid, and neurotransmitter synthesis pathways and calculated as the average percentage of ^13^C-labeled isotopologues (m+1 + m+2 … + m+n) of metabolites within each pathway relative to the ^13^C enrichment across all other pathways. For volcano plot, principal component analysis, fold enrichment plot and pathway impact analyses, the online tool Metaboanalyst (https://www.metaboanalyst.ca) was used. For pathway impact analysis, data were filtered using interquartile range (IQR) function and scaled using auto-scaling, in which mean-centered values were divided by the standard deviation of each variable. Global test’ and ‘Relative Betweenness Centrality’ parameters were used to determine metabolic pathway ‘hub’ importance.

### 4.10. Animal Cognitive Testing

Cognitive function was tested in WT and 5XFAD mice using a Morris water maze as previously described [53]. Trials were performed in a black pool (diameter 121.5 cm) filled with opaque water with a temperature of 22 °C and lighting between 41.0 and 43.0 lux. Mice were individually housed and allowed to acclimate to cages for 30 min prior to trial start. Mice were given two sessions per day, and each session consisted of two trials. Each session was separated by 3 h, and each trial by 10 min. Over a 4-day span, mice were trained during “Hidden Platform” trials to locate an “escape” platform (diameter 10 cm) submerged just under the surface of the water in the “target” quadrant. “Hidden Platform” trials lasted 60 sec, or until the mouse located the platform and remained on it for 3 sec. The drop location (where the mice were placed into the maze) was randomized for each trial, and never included a location within the “target” quadrant. For all consecutive “Hidden Platform” trials, the platform location was not changed. Visual cues (38 × 38 cm) were placed at the borders of each quadrant for reference. Spatial memory was assessed 72 h following completion of the “Hidden Platform” trials. During the “Probe” trial, the hidden platform was removed, and spatial memory was assessed by measuring cumulative distance from the platform location. Mice then received 2 more days of “Hidden Platform” training followed by a second “Probe.” Upon completion of the final “Probe” trial, mice received 2 “Visible Platform” trials with a colored flag (height of 18 cm) cue placed on the platform. All trials were recorded with a camera and analyzed using Ethovision XT (Noldus Information Technology, Leesburg, VA, USA).

### 4.11. Statistical Analysis

All data are expressed as mean values +/− standard error. Comparisons between two groups were analyzed by unpaired, two-tailed *t*-test or multiple unpaired, two-tailed *t*-tests (one per row) for comparisons with multiple isotopologues. Multiple time points (animal cognitive testing) were analyzed using ANOVAs or repeated measures ANOVA (time × groups). Statistical significance was determined using an error probability level of *p* < 0.05 corrected using the two-stage linear step-up procedure of Benjamini, Krieger and Yekutieli, with Q = 1%. Statistical details can be found in figure legends for each figure, all statistical analyses were calculated using GraphPad Prism 8 software (GraphPad Software, San Diego, CA, USA).

## 5. Conclusions

In conclusion, using an oral gavage to administer stable isotope tracers in mice is an effective tool for studying metabolism in vivo with broad applicability. We demonstrate a detailed time course for a number of metabolites and metabolic pathways following [U-^13^C] glucose delivery via oral gavage that shows sufficient incorporation of ^13^C into major metabolically active organs. We caution that these data be used as a starting point—a recommendation to guide future study designs. Many aspects of experimental conditions (phenotype, pharmacological intervention, and tracer selection) can greatly affect metabolite turnover rate and isotopic distribution, and researchers should carefully select parameters with these factors in mind. Future experiments should include gavage of other stable isotope tracers such as ^13^C- or ^15^N-glutamine and other amino acids, ^13^C-ketones, and ^13^C-fatty acids to trace their distribution and detail their time course. Finally, this method can be easily adapted further with expanded time points and sequential gavages to determine flux analyses and nutrient turnover rate of glucose and other tracers.

## Supplementary Materials

The following figures and file are available online at https://www.mdpi.com/2218-1989/10/12/501/s1: Figure S1: Plasma ^13^C enrichment in metabolites over time. Figure S2: Liver glycogen flux from ^13^C glucose. Supplementary File S1. Raw data as relative abundance (natural abundance stripped) and fractional enrichment.
