# Supplementary material for: Oral Gavage Delivery of Stable Isotope Tracer for In Vivo Metabolomics
Source: Metabolites. 2020 Dec 8;10(12):501. doi: 10.3390/metabo10120501 (PMC7764755; doi:10.3390/metabo10120501)
Supplement: Supplementary file 1 [file metabolites-10-00501-s001.zip › Supplementary Material/Supplementary Figures.pdf]

metabolites

MDPI

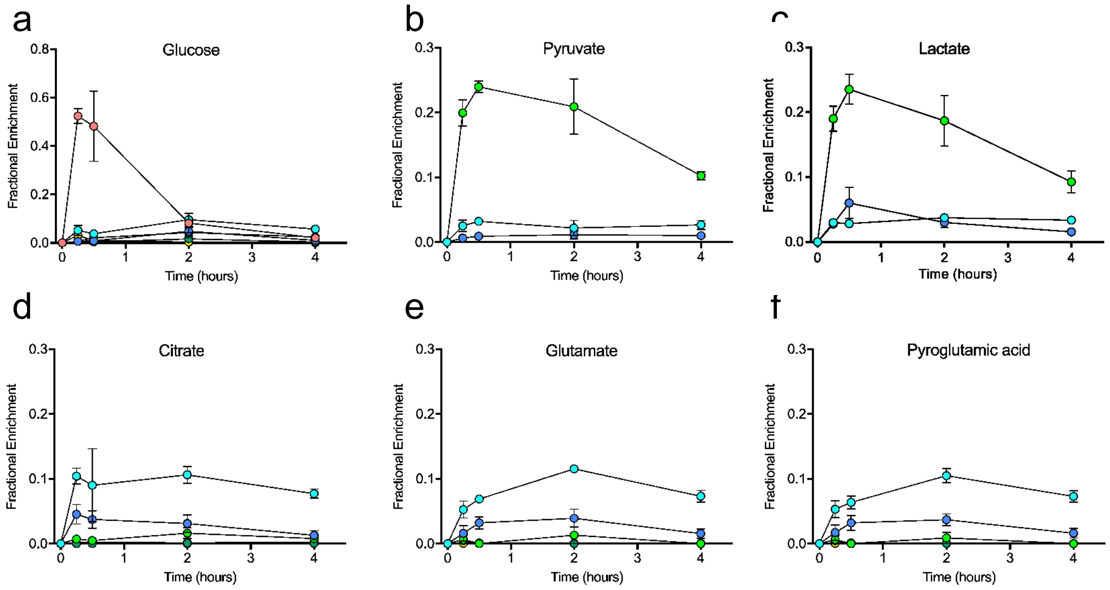

Figure S1. Enrichment of ^{13}C from glucose in plasma metabolites. Time course of labeled isotopologues from plasma at 15 min, 30 min, 2 h, and 4 h post-gavage. (**a**–**f**) Fractional enrichment of glucose (**a**), pyruvate (**b**), lactate (**c**), citrate (**d**), Glu (**e**), and pyroglutamic acid (**f**). Values shown are mean ± SEM (n = 2–5).

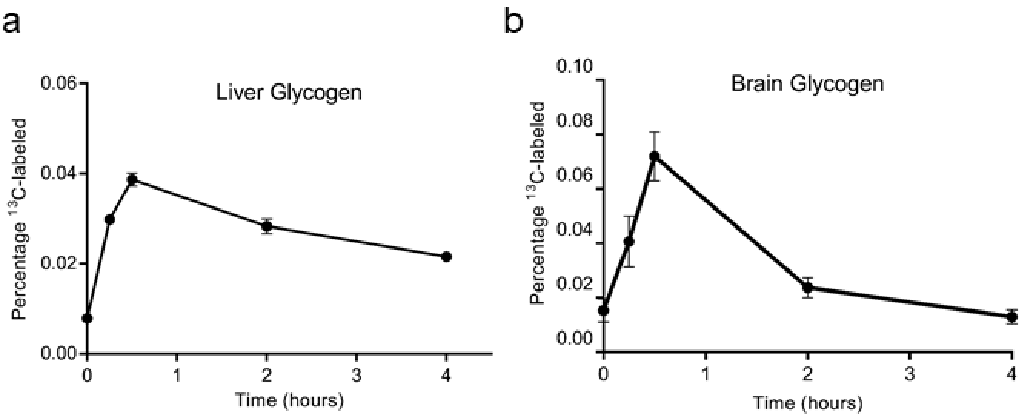

Figure S2. [U-^{13}C] Glucose incorporation into liver glycogen. Time course showing percentage of [U-^{13}C] glucose enrichment into liver glycogen (**a**) and brain glycogen (**b**) at 15 min, 30 min, 2 h, and 4 h post-gavage. Values shown are mean ± SEM (n = 3–5).

© 2020 by the authors. Submitted for possible open access publication under the terms and conditions of the Creative Commons Attribution (CC BY) license (http://creativecommons.org/licenses/by/4.0/).
